# Supplementary material for: Are carbon emissions trading and green financial instruments synergistic? -Comprehensive quantitative research based on content analysis
Source: PLoS One. 2024 Mar 7;19(3):e0298601. doi: 10.1371/journal.pone.0298601 (PMC10919601; doi:10.1371/journal.pone.0298601)
Supplement: S1 Data — (ZIP) [file pone.0298601.s001.zip › Data/地级市Z_F工作报告.zip!/╣π╬≈/╬α╓▌╩╨/└┤╫╘╣1⁄2╓┌║┼í╛┬φ┐╦╩2╛▌═°í┐_2018.docx]

各位代表：

现在，我代表市人民政府向大会作政府工作报告，请予审议，并请市政协各位委员提出意见。

一、2017年工作回顾

刚刚过去的一年，面对错综复杂的国内外经济形势和我市经济发展遇到的困难挑战，在自治区党委、政府和市委的正确领导下，我们深入学习贯彻党的十九大精神和习近平总书记视察广西时的重要指示，贯彻新发展理念，坚持稳中求进工作总基调，以突出经济运行过程管理及“三年一工程”活动为抓手，统筹抓好稳增长、促改革、调结构、惠民生、防风险各项工作，有力促进了经济社会发展总体稳定、稳中提质、稳中趋好。

（一）全力以赴稳增长，经济基本面持续向好。坚决贯彻落实中央和自治区各项稳增长政策措施，坚持精准施策、多点发力，强化三次产业协同拉动，加快推动产业转型升级。主要经济指标稳中有进。初步预计，全市完成地区生产总值1335亿元，增长7.5%左右；规模以上工业增加值721亿元，增长7%左右；固定资产投资1332亿元，增长14%；社会消费品零售总额440亿元，增长11%。投资结构不断优化。安排重大项目250个，年度计划投资207亿元，实际完成投资236亿元。全年工业投资、制造业投资分别完成587亿元、510亿元。大力推动政府和社会资本合作，全年开工PPP及相关项目20个，民间投资占固定资产投资的68%、贡献率提高到65%。工业企业效益整体回升。预计全市规模以上工业企业主营业务收入、利润总额保持两位数增长，产品产销率达97%；停产和负增长的规上工业企业比例从上年的29.8%下降到15.4%，为四年来同期最低。现代特色农业加快发展。全市种植富硒水稻3.5万亩，新增茶园面积8000亩，果树种植总面积超过78万亩，蜂群总数居全区第一，全市获得自治区畜禽现代生态养殖认证的规模养殖场98个。大力推进现代特色农业示范区建设，全市获认定的自治区级示范区7个，各级示范区经营总收入超过25亿元。服务业发展成效显著。加大对重点服务业企业扶持力度，新入库三产限上企业197家。大力实施品牌提升战略，新增18个广西名牌产品、3家“广西老字号”企业，2家企业被确定为广西商标品牌战略实施示范企业。消费市场保持较快增长，一批城市综合体成为拉动消费主力军。多举措推动电商发展，电子商务交易额193亿元，增长75.7%。加快发展旅游业，成功举办第二届广西岭南风情旅游文化周活动，新增5个国家4A级旅游景区和一批自治区级旅游示范区、星级乡村旅游点，蒙山县将获评为广西特色旅游名县。预计全年第三产业增加值增长10%，对经济增长的贡献率由上年的29.3%提升到36.5%。

（二）大力推动创新发展，经济发展质量持续提升。以创新引领转型升级，加快经济发展动力变革。创新驱动发展战略深入实施。高新技术产业完成投资82亿元，增长25.6%。大力推进梧州高新区创建国家级高新区，六堡茶种质创新与综合利用、天然药物创新制剂工程研究中心被确定为自治区级工程研究中心，梧州制药“广西三七综合利用技术重点实验室”成为我市首个获认定的广西重点实验室。扎实推进“双创”工作，建成国家级众创空间1家、国家级星创天地2家、自治区级星创天地2家、自治区级孵化器2家。传统产业加快转型升级。积极帮助金海、永达、鑫峰等不锈钢企业改造升级，促进整改停产企业全面恢复生产。狠抓钛白、陶瓷、纺织服装等传统产业技术改造，一批企业重新焕发生机，全年完成技改投资407亿元。新兴产业加快成长。加快推进稀土新材料产业园、静脉产业园等重大项目，新华大容量高品质电池、鼎立高性能钕铁硼永磁材料等项目实现投产。银行业支持实体经济力度加大，预计全市年末各项贷款余额787亿元，比年初增加65亿元。

（三）不断深化改革扩大开放，发展活力持续释放。统筹抓好各项改革试点工作，努力拓展开放领域、提高合作水平。供给侧结构性改革扎实推进。认真落实“三去一降一补”政策措施，采取扩大电力直接交易范围、扩大“惠企贷”受益面等举措降低企业综合成本，帮助企业减负超过7亿元，全市商品房消化周期降至11个月左右。积极防范金融风险，实现银行业不良贷款余额和不良贷款率“双下降”。完成33亿元政府性债务置换，政府性债务风险处于可控水平。重点领域和关键环节改革不断深化。深入推进“放管服”改革，取消调整行政许可事项312项，完成市行政审批局组建并挂牌运行，将26个部门224项行政审批事项划转至行政审批局集中办理，实现了“一枚印章管审批”，网上办事大厅上线运行。扎实推进商事制度改革，全市新增市场主体1.9万户，注册资本257亿元。国企国资、不动产登记、农村土地确权登记颁证、国有林场、供销社等改革稳步推进，长洲区农村集体资产股份权能改革成为全区典型。开放合作领域进一步延伸。主动对接和参与珠江—西江经济带、粤港澳大湾区、粤桂黔高铁经济带建设，成功举办西江经济带城市共同体及市长联席会第二次会议暨西江经济发展论坛，同广州等城市举办了市长联席会议，与广佛肇云茂等城市的务实合作深入推进。第十四届梧州宝石节取得圆满成功。粤桂合作特别试验区建设全面提速。加快推动试验区“1+3配套文件”落地，人事管理体制、行政审批、工业用地市场化配置、增量配电业务等改革稳步推进。深化投融资机制创新，发起3支产业基金规模60亿元，融资到位资金18亿元。云榄风力发电机、锂霸动力电池等6个项目投产试运营，国光产业基地、中兴广西云数据中心等14个项目实现开工建设。试验区成为广西首批CEPA先行先试示范基地。预计试验区工业总产值和固定资产投资分别增长14%、30%，“三年打基础”目标基本实现。外向型经济加快发展。精心组织重大招商活动，成功引进一批战略投资者，正威宝玉石文化创意产业园、毅马年产8万吨五金制品、欧神诺陶瓷等一批项目落户梧州，全市招商引资区外到位资金670亿元，增长8.6%。多措并举力推外贸恢复性增长，扭转了外贸进出口连续33个月下降的颓势，预计全年外贸进出口总额增长34%。

（四）加大统筹城乡发展力度，新型城镇化持续推进。大力推进以人为核心的新型城镇化，加快完善城乡基础设施，城乡品质不断提升。城市建设三年提升工程成效明显。组织实施基础设施、新区建设、公共服务提升等工程，全年完成投资77亿元，三年累计完成投资252亿元。加快中心城区基础设施建设，完成了一批城市主干道改造提升工程，西江三桥建成通车，龙圩综合客运枢纽大楼主体工程完工。加快苍海新区和红岭新区开发建设，新区路网、景观等基础设施项目加快推进，苍海小学、恒大绿洲、彰泰玫瑰园等项目顺利推进。大力实施棚户区改造工程，开工建设9446户（套），完成投资12亿元。立体交通格局进一步完善。梧州至柳州高速公路、市环城高速公路省际通道建成通车，贺州至巴马高速公路蒙山段、荔浦至玉林高速公路开工建设，西江机场场道工程及航站楼、航管楼主体工程进展顺利，207国道东绕城过境公路、龙圩经大坡至新地公路、苍梧梨埠至昭平马江公路、梧州至藤县藤州镇公路、蒙山至金田公路等项目积极推进。铁路运输能力不断释放，新增19对途经梧州南站动车，铁路客运量增长17.3%、货运量增长59%。新竣工一批码头、泊位工程，西江运能不断提升，梧州港吞吐能力达到5600万吨，预计全年实际完成港口货物吞吐量3620万吨，集装箱吞吐量64万标准箱，分别增长7%和22.3%。供电保障能力有新提升。合理布局供电设施，新建扶典220千伏、塘源110千伏等送变电工程，推动试验区、龙圩区等重点区域的电网升级。推动新能源项目落地，静脉产业园生活垃圾焚烧发电、金色能源公司100兆瓦光伏发电、苍梧岭脚风力发电等项目稳步推进。县域城镇化建设取得新成效。苍梧新县城建设取得重大进展，县委、县政府进驻新县城办公。岑溪市、藤县和蒙山县产城融合发展迈出新步伐，自治区百镇示范建设工程扎实推进，苍梧县六堡特色小镇入选国家第二批特色小镇名单。

（五）始终坚持绿色发展，生态环境持续向美。牢固树立绿色发展理念，大力发展生态经济，加大生态环境保护治理力度。循环经济产业不断壮大。继续做长延伸循环经济产业链条，国家“城市矿产”示范基地通过国家终期验收，循环经济产业园区获“全国循环经济优秀园区”称号，预计园区完成工业总产值425亿元。环境保护力度加大。完成植树造林22万亩，全市森林覆盖率达75.9%，顺利通过“国家森林城市”复查。落实河长制和水污染防治行动计划，全市25个地表水监测断面的水质达标率保持100%，集中式饮用水水源地水质达标率94.7%，空气质量优良率90.4%，西江流域“一干七支”生态建设试点稳步推进。积极开展宝石产业综合整治，努力解决群众反映强烈的宝石加工污染问题。“美丽梧州”乡村建设活动成效显著。大力推进“宜居”乡村建设，全市累计有314个行政村完成“六有”标准规范化建设、411个行政村建成村级公共服务中心。实施农村生活垃圾治理两年攻坚行动，建成垃圾处理设施88个。开展农村环境综合整治工程，建成农村生活污水处理设施99座（套）。抓好自治区级生态县、镇、村创建工作，蒙山县荣获自治区级生态县称号，全市18个乡镇和12个村分别获得自治区级生态乡镇、生态村称号。

（六）大力保障和改善民生，民生福祉持续增进。始终坚持以人民为中心，倾力办好各项民生实事。民生保障水平进一步提升。财政支出83%以上用于民生，11项为民办实事工程完成投资46亿元。预计全市城乡居民收入增长8%。城乡新增就业8.6万人次，城镇登记失业率控制在4.5%以内。调整提高城乡低保、特困人员供养、退休人员养老金标准共惠及38万人，基本养老、基本医疗保险参保人数分别达162万人和324万人。全面完成脱贫目标任务。筹措扶贫专项资金9亿元，发放扶贫贷款13亿元、贴息5900多万元，投入4亿元开展扶贫基础设施建设，投入4900多万元扶持产业发展。抓好9个易地扶贫搬迁项目，累计完成投资4亿元。全市54个贫困村和蒙山县达到脱贫摘帽标准，预计实现6.5万人脱贫。“第一公里·桥”惠民工程初战告捷。聚焦影响农村群众出行“第一公里”的安全和通畅问题，全年改建新建桥梁（涵）320座，完成投资7200万元，惠及群众近100万人，“第一公里·桥”成为“连心桥”“暖心桥”，赢得老百姓广泛赞誉。社会事业全面进步。坚持优先发展教育事业，学前三年毛入园率、义务教育巩固率、高中阶段毛入学率分别为85.4%、97.7%、89.5%；高考一本上线1871人，创历史新高，本科、专科上线率均排全区前列；校园及周边治安综合治理工作成效显著，我市成为全区首个国家级校园安全教育试验区。稳步推进公立医院综合改革，全面实施分级诊疗制度，5家三级医院组建了医联体，县级医院组建了4个医共体，实现药品零差率销售全覆盖。完善公共文化服务体系，新建文化基础设施72个、村级公共服务中心50个；成功举办第三届广西（梧州）粤剧节，推出反映“岭南记忆”的精品粤剧《风雨骑楼》，电影《天各一方》荣获广西精神文化建设“五个一工程”奖、第八届广西文艺创作铜鼓奖。梧州市及蒙山县分别荣获第七轮自治区文明城市称号，市审计局荣获全国文明单位称号。加快推动体育事业发展，成功举办或承办全国青少年足球锦标赛、“一带一路”国际（梧州）青少年足球邀请赛。优化养老服务供给，“云家庭”服务中心覆盖3个城区和藤县，“互联网+社区居家养老”模式入选全国养老优秀案例。社会大局和谐稳定。“七五”普法深入开展，法律服务“五张网络”模式获自治区肯定并在全区推广。平安梧州建设持续深化，组织开展“神剑”系列专项行动“五大战役”，严厉打击危害社会治安的违法犯罪活动，社会治安防控体系建设水平不断提升，信访维稳、应急管理、安全生产、食品药品监管等工作不断加强，群众安全感排名位居全区前列，万秀区荣获全国平安建设先进县（市、区）称号，蒙山县、长洲区荣获平安广西建设先进县（市、区）称号。

（七）着力强化责任担当，政府自身建设持续加强。学习贯彻党的十九大精神，推进“两学一做”学习教育常态化、制度化，大力整治为官不为和“庸懒散”现象，打造强执行敢担当善作为的公务员队伍，营造风清气正的干事创业氛围。加快法治政府建设，把重大行政决策合法性审查延伸到政府各部门，实行政府规范性文件管理“三统一”制度，实现政府部门权责清单“两单融合”。建立健全政府立法机制，组织起草《梧州市停车场建设和管理条例（草案）》，制定首部政府规章《梧州市拟定地方性法规草案和制定政府规章程序规定》。自觉接受人大及其常委会法律监督和工作监督，主动接受政协民主监督，积极采纳人大代表、政协委员的意见建议，人大代表建议和政协委员提案办结率100%。认真听取各民主党派、工商联、无党派人士和各人民团体的意见建议。深入贯彻落实中央八项规定精神，大力压减财政一般性支出，加强政府投资项目评审管理。落实政府党组全面从严治党主体责任，加强行政监察和审计监督，抓好重要部门、重点岗位、重点领域的廉政风险防控，反腐败斗争保持高压态势，政府系统党风廉政建设取得新成效。

一年来，国防动员、民兵预备役、双拥创建、人民防空、消防、防震减灾等工作扎实有效开展，编制、民族宗教、外事侨务、港澳台、统计、农机、物价等工作取得新进步，工会、共青团、妇女儿童、残联、科协、社会科学、慈善、档案、地方志等工作取得新成效，中直、区直驻梧各单位为全市经济社会发展做出重要贡献。

各位代表，回顾过去一年的工作，我们取得的成绩来之不易，这是自治区党委、政府和市委正确领导的结果，是市人大和市政协监督支持的结果，是全市人民团结奋进、努力拼搏的结果。在此，我代表市人民政府，向全市各条战线的干部群众，向人大代表、政协委员，向各民主党派、工商联、无党派人士、人民团体，向离退休老领导、老同志，向驻梧部队、武警官兵、公安干警，向所有关心支持梧州改革发展的社会各界人士，表示崇高的敬意和衷心的感谢！

我们清醒地认识到，当前发展中还存在不少困难和问题，主要是：经济企稳向好的基础尚不牢固，稳增长的任务十分艰巨；产业结构不合理，制造业规模小、质量不高，企业创新能力不强；资金、土地等要素趋紧，资源环境约束加大，重大项目落地难问题不同程度存在；财政收入面临结构不优、税源不足、收支不平衡等困难，去年财政收入出现负增长；城乡基础设施、教育、医疗卫生、养老等领域仍存在不少短板；发展环境有待进一步优化，发展的紧迫感和干部作风建设有待进一步加强，等等。对此，我们将高度重视，采取更加有力的措施，努力加以解决。

二、2018年主要目标任务和重点工作安排

今年，是贯彻落实党的十九大精神的开局之年，是改革开放40周年，是决胜全面建成小康社会、实施“十三五”规划承上启下的关键一年，也是自治区成立60周年，做好今年工作意义重大。党的十九大作出了中国特色社会主义进入新时代的重大判断，提出了习近平新时代中国特色社会主义思想，明确了新时代的基本方略、奋斗目标、战略安排和工作布局，描绘了决胜全面建成小康社会、建设社会主义现代化强国的宏伟蓝图，为我们做好今年和今后一个时期的工作提供了根本遵循、指明了前进方向。我们必须准确把握党和国家发展新的历史方位、新的历史变化和新的任务要求，立足基本市情，深入谋划好新时代梧州的发展目标、发展思路、发展举措，以永不懈怠的精神状态和一往无前的奋斗姿态，担当新使命，展现新作为，扎实推动各项事业全面进步。

根据市委的决策部署，今年政府工作的总体要求是：全面贯彻党的十九大精神，以习近平新时代中国特色社会主义思想为指导，坚持稳中求进工作总基调，贯彻新发展理念，按照自治区第十一次党代会、市第十三次党代会及全市经济工作会议的安排部署，牢牢把握高质量发展这个根本要求，以推进供给侧结构性改革为主线，以做强制造业、做大服务业为重点，着力构建现代化经济体系，统筹做好稳增长、促改革、调结构、惠民生、防风险各项工作，加快推动质量变革、效率变革、动力变革，全面推进三大攻坚战，促进经济平稳健康发展和社会和谐稳定，为如期完成“三个实现”总体目标奠定更加坚实的基础。

全市经济社会发展的主要预期目标是：地区生产总值增长7.5%左右，规模以上工业增加值增长7.5%，第三产业增加值增长10%，固定资产投资增长12%，财政收入增长2%，社会消费品零售总额增长10%，外贸进出口总额增长6%，城乡居民人均可支配收入增长8%左右，节能降碳减排等约束性指标完成自治区下达目标任务。

为完成上述目标任务，重点抓好以下几项工作：

（一）大力发展实体经济，加快构建现代化经济体系。坚持把发展经济的着力点放在实体经济上，把提高供给质量作为主攻方向，加快推进经济结构战略性调整，努力推动高质量发展。

深化供给侧结构性改革。深入推进“三去一降一补”，着力在降成本中增强实体经济活力，在补短板中扩大有效投资。开展“降成本政策落实年”活动，落实自治区出台的降成本“41条”和新“28条”，建立降成本联合推进机制、成本负担监测机制和信息反馈机制，进一步降低企业综合成本。完善政府性融资担保体系，采取支持企业上市、发展债券融资、深化股权投资等措施，拓展企业直接融资渠道，破解企业融资难、融资贵的问题。畅通民间资金进入实体经济的渠道，放开基础设施、公共服务、生态环境等领域投资，力促民间投资增速及占比 “双提升”。继续对重点企业实行“一企一队、一企一策”上门服务，帮助企业按市场化方式努力破解高质量发展瓶颈。

着力做强制造业。大力推进工业转型升级，加快食品医药、林产林化、钛白、陶瓷等传统优势产业“二次创业”，推动再生资源、再生不锈钢向精深加工方向和产业链高端延伸，支持金海不锈钢、金茂钛白等企业加大技术改造和产品升级力度，力争全年完成技改投资270亿元。着力提升制造业整体竞争力，把存量做优、增量做大，支持中恒集团、神冠集团等企业发展壮大，重点推进中国—东盟环保技术和产业交流示范基地、再制造示范基地等基地型项目建设，推动沃特玛新能源汽车、中国电信创科中心等新兴产业项目开工建设，力争新增规上企业30家以上。持续推进产业园区提质扩容，加大对梧州高新区、循环经济产业园区、陶瓷产业园区、稀土新材料产业园区等重点产业园区基础设施和配套设施投入，开工建设和平重型起重机械生产、年产30万吨铝锭铝棒生态铝产业园、业进陶瓷等一批项目，力促毅马年产8万吨五金制品、锂离子电池自动化设备生产、欧神诺陶瓷等一批项目投产，力争园区工业总产值超过2400亿元。

加快做大服务业。实施“服务业百项重点工程”，推进粤桂试验区综合性服务业集聚区、市商贸物流园、苍海中央商务区等项目建设。充分挖掘消费潜力，改造提升老城区传统商圈，做优做强新城区新兴商圈，发展数字家庭、远程定制、体验分享等消费新模式。大力发展电子商务，推动毅德城广西电子商务示范基地、西江经济带（梧州）跨境电商产业园等聚集区开园运营，力争电子商务交易额达260亿元以上。以创建全域旅游示范区引领旅游业转型升级，加快“三总府”文化旅游博览园、袁崇焕故里、思良江乡村旅游示范点等景区景点建设，推动骑楼城、石表山创建国家5A级景区，争创国家4A级旅游景区2个、广西生态旅游示范区1家、星级乡村旅游区2家以上。大力发展健康养老产业，扎实推进岭南健康养生城前期工作，开工建设国家开放大学岭南老年学院。

规划建设西江南岸百里经济带。按照“统一规划、区域协同、绿色生态、要素融合、共建共享”的原则，以粤桂合作特别试验区的江南片区和社学片区、苍海新区、岭南健康养生城、循环经济产业园区、临港经济区、藤县陶瓷园区等为主体，规划建设东起粤桂省界、西至藤县县城东的西江南岸百里经济带，强化基础设施互联互通，强化政策资源和公共服务设施共享，强化特色化差异化发展，大力培育发展新经济新动能，努力打造成为西江黄金水道上向东开放龙头城市的战略支撑带和引领全市经济发展“三大变革”的重要引擎，在广西与粤港澳经济合作中发挥重要作用。

（二）大力实施乡村振兴战略，加快推进农业农村现代化。按照“产业兴旺、生态宜居、乡风文明、治理有效、生活富裕”的总要求，建立健全城乡融合发展体制机制和政策体系，做优现代特色农业，不断提升农业农村发展水平。

着力构建现代农业产业体系。坚持质量兴农、绿色兴农，加快推进农业由增产导向转向提质导向。坚守耕地保护红线，确保粮食生产稳定。围绕构建“水陆空”三位一体立体农业格局，大力发展“富硒+”农业，做大做强名优果蔬、生猪、三黄鸡、草食动物、网箱鱼、蜜蜂等种养业，重点推进温氏农牧养殖、岑溪汉世伟现代生猪养殖、超级富硒水稻试验田等项目建设，打造现代农业产业集群。推进六堡茶产业升级发展，发起六堡茶产业基金，推动六堡茶产业园建设，新增标准化茶园1万亩以上。培育新型农业经营主体，力争建成市级农业产业化重点龙头企业3家以上、农民合作社示范社20家以上、土地股份合作社50家以上，新增家庭农场50家以上。推动现代农业示范区建设增点扩面提质升级，创建核心示范区5个以上、示范区8个以上、示范园13个以上。积极打造田园综合体，争取每个县（市、区）至少创建1个核心示范区、2个示范区，每个乡镇至少创建1个示范园。推广“微生物+”畜禽现代生态养殖模式，生态养殖场认证率达80%以上。

推动农村一二三产融合发展。大力发展特色农业、精品农业，加强品牌营销，打造一批在区内外叫得响、销售好的农业品牌。鼓励农业企业建设仓储、预冷库和物流基地，发展冷链物流、电子商务、连锁经营等业态，推动农业与服务业融合发展。以六堡特色小镇为载体，加快茶旅融合发展，积极培育古茶村、茶民宿等新业态，打造一二三产融合发展示范项目。以主办“2018北京两展一节”为契机，不断扩大六堡茶影响力。加快发展农村电子商务，打造“一县一品”农村电商品牌，新增农村电商服务点100个以上，力争农村电子商务交易额增长40%。

坚决打赢脱贫攻坚战。坚持把脱贫攻坚作为头等大事来抓，深入推进“七个一批”“十大行动”，确保完成自治区下达的脱贫摘帽任务。推进脱贫重点领域攻坚，统筹道路、饮水、农村电网等基础设施建设。大力发展特色种植、生态养殖、乡村旅游等产业，促进贫困村农民增收。实施11个易地扶贫搬迁项目，完成投资2.5亿元，搬迁4000人。盘活村级资源，增强“造血”功能，培育壮大村级集体经济。加快创建自治区示范性农民工创业园，加强农村贫困劳动力职业技能培训，促进农民创业。坚持专项扶贫、行业扶贫、社会扶贫并举，加快形成大扶贫格局。

（三）全力推进改革创新，不断增强发展动力活力。坚持把改革创新贯穿于经济社会发展各领域，把发展基点放在改革创新上，在改革创新中赢得后发优势。

大力推动创新驱动发展。支持梧州高新区创建国家级高新区，大力推进高新区升级“836”计划。提升传统优势产业创新能力，重点在食品医药、陶瓷建材、再生资源等领域开展技术研发，推动产业链向中高端迈进。加快科技创新平台建设，创建4个以上自治区级工程技术研究中心、重点实验室。加强公共检测平台建设，建成西江食品医药检验中心，筹建国家黑茶（六堡茶）产品质量监督检验中心。健全科技企业孵化器和众创空间梯队培育机制，建设2家以上“双创”基地。深入实施发明专利双倍增计划，促进发明专利不断推陈出新。加快培育高新技术企业，力争高新技术企业达到50家以上。

进一步深化“放管服”改革。加大简政放权力度，继续削减行政审批事项，规范行政审批中介服务，简化投资项目报建审批，进一步清理规范涉企收费。深入推进商事制度改革，全面推行“多证合一”“证照分离”、市场主体电子营业执照和登记全程电子化。推进政府监管体制改革，推行“双随机一公开”抽查机制，不断提高事中事后监管实效。充分发挥市行政审批局作用，优化行政许可集中审批系统功能，推动“一枚公章管审批”机制高效运转。完善市县乡村四级政务服务体系，推进市政务服务中心标准化建设，深化“互联网+政务服务”，打造24小时“不打烊”网上政府。

统筹推进其他重点改革事项。推进国企国资改革，加快市属国有企业整合重组与转型升级。深化投融资体制改革，建立完善PPP有效推进机制。加快增量配电业务改革，扩大直供电规模和范围。推广长洲区农村集体资产股份权能改革经验，扩大改革试点面。做好农村土地承包经营权确权登记颁证和农村金融、水利、林业、供销等方面改革。统筹推进文化体制、医药卫生体制、统计体制改革，深化城市管理执法体制改革、事业单位分类改革等。

（四）深入实施“东向”战略，以向东开放带动全面开放。坚持以粤港澳为主攻方向，加快融入珠三角，对接北部湾，积极参与区域产业分工，发展更高层次的开放型经济。

大力推进区域合作。积极融入“一带一路”，全面参与珠江—西江经济带和粤桂黔高铁经济带建设。对接粤港澳大湾区，主动配合自治区争取粤港澳大湾区延伸到梧州，参与“大湾区”产业链分工合作。务实推进与广佛肇云茂等城市的战略合作，不断提升全面互联互通水平，探索建立更加紧密的基础共建、产业共兴、利益共享等机制。积极参加中国—东盟博览会、泛珠三角经贸洽谈会等重大经贸活动，办好第十五届梧州宝石节、西江经济带城市共同体及市长联席会议第三次会议暨西江经济发展论坛。

加快粤桂合作特别试验区建设。全面推动试验区“1+3配套文件”落实。推进试验区路网管网、供水排水等基础设施建设，加快粤桂总部基地、棚户区改造等配套设施建设。加快产业集聚发展，推进国光产业基地、中盟产业园、台湾高新科技产业园等项目建设，争取红牌智慧家电、东雷环保锂电池、丰歌低碳绿屋建造等项目竣工投产。加快CEPA先行先试示范基地、两广金融改革创新综合试验区、两广现代服务业聚集区、军民融合创新产业园建设，做实做细申报综合保税区前期工作。推动深圳国有资本参与试验区建设，促进粤桂股份制公司投入运营。力争全年完成工业总产值160亿元，固定资产投资100亿元，建成通用标准厂房面积30万平方米；完成招商引资区外到位资金90亿元，一批实体经济企业落地、开工建设，新增规模以上工业企业5家以上。

强力推进精准招商。主动对接世界500强、中国500强等大企业大集团，争取引进一批龙头企业。围绕做大做强支柱产业，着力引进产业关联性大、带动力强的上下游项目，加快培育壮大产业集群。进一步完善招商考评考核与激励约束机制，不断提高合同履约率、资金到位率和项目开工率。强化招商引资的主体责任，充分发挥各专业招商队和招商大使、招商专员、招商顾问的作用，力争全年招商引资区外到位资金达到720亿元以上。

大力发展外向型经济。全力推进第二轮“加工贸易倍增计划”，提升加工贸易集聚发展水平。支持外贸综合服务平台和出口基地建设，确保外贸进出口总额稳定增长。引进和培育知名电商企业、电商平台，努力实现电子商务跨越式发展。推进通关一体化改革，加快口岸“单一窗口”等通关便利化建设，推动赤水港扩大开放验收，做好内河口岸“单一窗口”建设。继续实施集装箱定期班轮航线补助政策，降低企业物流成本，提高梧州口岸竞争力。

（五）加快推进新型城镇化，推动城乡协调发展。注重发展壮大县级城市，积极培育中心镇、特色镇，加快形成分工有序、优势互补的空间格局，提高城镇化发展质量和水平。

进一步改善中心城市面貌。继续实施城市建设提升工程，加快推进重要片区、主要轴线、关键节点的城市设计，完善以快速路网、停车场为重点的基础设施，力争完成投资45亿元。大力推进城市新区开发建设，苍海新区重点推进苍海整体城镇化（一期）、岭南健康养生城、苍海旅游度假区、苍海文化中心、苍海水上乐园等项目建设；红岭新区重点推进城市路网、排水排污等基础设施建设，加大农贸市场、商业广场等配套设施建设力度。加快推进西江四桥等项目建设，力争环城高速公路、龙圩综合客运枢纽建成投入使用，西江机场实现试通航；加快打通市区“断头路”、修复破损路，畅通市区交通“微循环”。实施棚户区改造三年规划，力争全年开工10627户（套）、基本建成2034户（套）。优化完善公交线路、站点和运力结构，推动市县公交“一卡通”。加快建设城区智慧停车场，切实解决停车难问题。实施苍海大道、新兴三路、奥奇丽路等城市主干道和重要出入口绿化、美化、亮化提升工程，规划建设市区南岸山体夜景新景观。

加大力度打造特色城镇群。加快苍梧新县城建设，抓好道路、供排水等基础设施以及公共服务设施建设。支持岑溪、藤县按中等城市标准加快县城建设，推进岑溪市新型城镇化试点县建设，推动藤县撤县设市。支持蒙山县按小城市标准提升规划建设水平，争创全国文明城市（县级）。实施“城镇扶持壮大工程”，完善小城镇基础设施和公共服务设施，抓好六堡特色小镇、长洲岛民俗风情旅游小镇、蒙山丝艺小镇建设，推进龙圩镇、旺甫镇、归义镇、太平镇、陈塘镇等重点镇建设，推动水汶镇、新圩镇等自治区百镇建设示范工程建设。扎实推进户籍制度改革，全面放开小城镇落户条件。加强小城镇环境综合整治力度，重点整治镇容镇貌“破、旧、乱”现象，促进城镇面貌和环境质量全面提升。

加强城乡基础设施建设。推进洛湛铁路电气化改造、梧州至信都高速公路等项目前期工作，加快贺州至巴马高速公路蒙山段、荔浦至玉林高速公路、梧州至藤县藤州公路、蒙山至金秀二级公路、苍梧梨埠至昭平马江公路、207国道东绕城过境公路等公路建设，争取贺州—梧州—玉林城际铁路开工、岑罗铁路项目复工建设。实施农田水利、水库除险加固、中小河流治理工程，抓好高旺、塘源、三龙防洪堤等工程建设。推动一批220千伏送变电工程竣工，加快小水电扩容提质和农村电网改造。围绕镇镇通二级路实施一批农村道路项目，加快推进“四好农村路”建设。抓好一批镇级污水处理项目建设，推动村级网络升级。

加快县域经济发展。落实自治区县域经济发展三年行动计划，开展县域经济发展“双千”项目大会战，计划安排项目1000个以上，总投资达1000亿元，重点推进一批产业项目、重大基础设施项目和公共服务设施项目，夯实县域经济发展基础。着力推进特色产业发展，力争每个县（市、区）打造2—3个特色产业集群，其中苍梧县重点抓好六堡茶加工、林产品精深加工等特色产业，岑溪市重点抓好稀土新材料、石材加工等特色产业，藤县重点抓好建筑陶瓷、钛白等特色产业，蒙山县重点抓好茧丝绸精深加工等特色产业。

（六）着力推进绿色低碳发展，加快建设美丽梧州。牢固树立“绿水青山就是金山银山”的发展理念，坚定不移走生态优先、绿色发展之路，努力提供更多优质生态产品，不断满足人民群众对优美生态环境的需要。

推进主体功能区建设。加大生态系统保护力度，加强重点生态功能区建设管护，确保森林覆盖率稳定在75%左右。抓好西江流域水生态保护与综合整治，构筑西江绿色长廊。加快蒙山县国家重点生态功能区建设。积极推进江南国家森林公园、太平狮山公园、飞龙湖国家森林公园和苍海国家湿地公园建设。建立健全生态保护优先的绩效考核评价机制，推动发展方式转变。

解决环境突出问题。加快推进市第一污水处理厂提标改造、市第三污水处理厂、城市新区“雨污分离”工程建设，持续推进宝石产业综合整治，加强危险废物处置能力和监测能力建设。深入实施“大气十条”，重点开展工业废气、城市扬尘、餐饮油烟等专项整治，确保空气质量优良率达到91%以上。深入实施“水十条”，推进市、县两级重点水源地保护区整治，全面推行河长制，抓好西江、浔江、桂江等江河湖库保护和治理，确保江河水质达标率保持在96%以上。深入实施“土十条”，开展土壤污染详查，抓好受污染基本农田、工业场地土壤治理与修复，加大农业面源污染防治力度。推进梧州“智慧环保”平台建设，加强对环境质量及污染源排放情况实时监控、动态分析，构建依托大数据精准治理的污染防治攻坚新格局。

大力发展生态经济。发展生态工业，推动不锈钢制品、陶瓷建材等传统产业清洁化生产，加快构建节能环保、新能源、新材料等新型生态工业产业链。实施生态种养工程，推动果蔬茶叶、畜禽养殖等生态化改造。实施林业“金山银山”工程，做大林下经济，力争林业生态经济完成产值600亿元。深入推进国家循环经济示范城市建设，巩固国家“城市矿产”示范基地创建成果。统筹推进节能降碳，加强能源消耗总量和强度“双控”，有效控制温室气体排放。

加快美丽乡村建设。深入推进宜居乡村“产业富民”“服务惠民”“基础便民”专项活动，启动“幸福乡村”建设活动。实施农村环境改善提升行动，扎实推进农村垃圾、污水整治和“厕所革命”，不断改善农村人居环境。推动美丽乡村建设与脱贫攻坚、生态旅游等有机结合，打造宜居宜业宜游的美丽乡村示范带。

（七）坚持在发展中保障和改善民生，不断提升群众幸福指数。始终把人民对美好生活的向往作为奋斗目标，以更大的决心、更大的力度保障和改善民生，不断增强人民群众的获得感、幸福感。

优先发展教育事业。深入实施第三期学前教育行动计划，推进学前教育普惠优质发展，建立健全幼儿园规范办园行为督导机制。大力推进义务教育均衡发展，力促苍梧县、藤县、龙圩区通过自治区督导评估，长洲区通过国家认定。加快构建小学、初中、高中教育质量监控体系，全面提升教育质量。续建2所、改扩建17所普通高中，争取提前三年完成高中阶段毛入学率达到90%的目标。加快中等职业学校布局调整，深化县级职业教育综合改革，推进集团化办学。发展特殊教育，加快县级特殊教育学校建设。加强师德师风建设，全面提升教师队伍素质。全力支持梧州学院建设特色鲜明的应用型高水平大学，加快梧州职业学院发展。

大力发展文化体育事业和文化体育产业。深入实施文化惠民工程，丰富群众性文化活动。加快县（市）图书馆、农家（社区）书屋等项目建设。深入开展历史文化名城、全国文明城市创建活动。大力发展文化产业，引导鼓励广大文艺工作者创作一批反映新时代特点、富有梧州特色的文艺作品。深入开展“扫黄打非”工作，加强文化市场监督。加大基层公共体育设施建设力度，组织开展群众体育健身活动，积极备战广西第十四届运动会，推进全民健身与全民健康深度融合发展。

提高就业质量和人民收入水平。认真落实自治区就业创业工作新“11条”措施，努力实现更高质量和更充分就业。鼓励大众创业万众创新，加快创业孵化基地、农民工创业园等平台建设，以创业带动就业。统筹做好高校毕业生、贫困家庭成员、就业困难人员等重点群体就业工作，力争城镇新增就业2.5万人，农村劳动力转移就业4.5万人次，城镇登记失业率控制在4.5%以内。深入实施城乡居民收入倍增计划，积极拓宽居民收入渠道，努力实现居民收入与经济增长同步。

健全社会保障体系。降低个人医疗自费比例，实施全民参保计划，推进城乡居民基本医疗保险整合工作，统筹失业、工伤、养老、医疗保险，实现全市社保“一卡通”。加强城乡社会救助体系建设，完善最低生活保障制度。全市农村低保平均保障标准达到人均3400元以上。完善社会福利、慈善事业、优抚安置等制度，健全农村留守儿童、妇女、老人和残疾人关爱保障机制。严厉打击劳动领域违法犯罪行为，全面治理拖欠农民工工资问题。

加快建设健康梧州。实施《“健康梧州2030”规划》，全面启动健康梧州建设。持续深化公立医院改革，全面推进分级诊疗工作，建立覆盖城乡的基本医疗卫生制度。实施国家信息惠民示范城市“智慧医疗”工程，加快推进市级区域人口健康信息平台和居民健康卡建设，实现家庭医生签约服务全覆盖。加快市工人医院、儿童医院等改扩建项目建设。完善卫生计生人才激励机制，改善乡镇卫生院就医环境，提高乡镇卫生院医疗水平。落实计划生育目标管理责任制，促进生育政策与经济社会发展政策配套衔接。做好艾滋病、慢性病、传染病等重大疾病的宣传和防治。创建国家社会办中医试点示范市。深入开展食品安全示范城市创建工作，加强食品药品安全整治，严防严管严控食品药品安全风险，不断提高食品安全治理能力和保障水平。着力构建养老、孝老、敬老政策体系和社会环境，推进医养融合，加快发展健康养老产业。

各位代表，保障和改善民生没有终点站，只有持续不断的新起点。今年我们将继续集中财力、整合资源，实施一批为民办实事项目，重点做好“第一公里·桥”惠民工程、就业创业、教育文化、医疗卫生、养老服务、百姓安居等民生实事，预计总投资超过50亿元，让新时代的改革发展成果更多更公平地惠及全市人民，让新时代的惠民政策转化为群众实实在在的获得感！

（八）加强和创新社会治理，构筑共建共治共享格局。加强社会治理制度建设，完善党委领导、政府负责、社会协同、公众参与、法治保障的社会治理体制，提高社会治理社会化、法治化、智能化、专业化水平。

创新社会治理。深入推进依法治市，加快法治梧州建设。加强社区治理体系建设，推动社会治理重心向基层下移，发挥社会组织作用，实现政府治理和社会调节、居民自治良性互动。全面开展“七五”普法，深入推进“法律六进”活动。强化守信激励和失信惩戒联动机制，建设诚信社会。创新信访工作体制机制，依法分类处理信访诉求，妥善化解社会矛盾，维护群众合法权益。

防范重大风险。加强政府债务管理，建立健全地方政府债务风险评估、预警机制和防范化解体系。牢牢守住不发生系统性金融风险的底线，做好金融领域风险防范和处置，维护良好的金融生态环境。认真落实安全生产“党政同责、一岗双责、失职追责”责任制，狠抓重点领域整治、隐患排查治理，守住安全生产底线，确保不发生重特大安全生产事故。深化“平安梧州”建设，严厉打击各类违法犯罪行为，健全立体化、数字化社会治安防控体系，增强突发公共事件应急预警处置能力，不断增强人民群众安全感。

加强双拥优抚安置工作，创新军民深度融合发展机制，大力支持国防和后备力量建设。支持工会、共青团、妇联、残联等群团组织建设。进一步做好民族宗教、外事侨务、保密、对台、统计、审计、地方志、物价、水文、气象、人防、移民、地震、农机、档案、老龄、机关事务等各项工作。

各位代表，做好今年工作，实现既定目标，必须加强政府自身建设。坚持以为民服务作为政府工作的出发点。深入推进“两学一做”学习教育常态化制度化，开展“不忘初心、牢记使命”主题教育，把人民群众所需所想所盼放在心上，推动服务向民生覆盖、资源向民生倾斜，使政府各项决策近民情、顺民意、贴民心。大力弘扬党的优良传统和作风，问政于民、问计于民、问需于民，把更多时间用在基层调研和现场办公上，把更多精力用在政策落实的“最后一公里”上，精准发力破解发展难题。坚持以依法行政作为政府工作的生命线。坚持用法治思维、法治方式履行职责，推动政府工作全面纳入法治轨道。自觉接受人大监督、政协民主监督和社会各界监督。认真办理人大代表建议和政协委员提案，虚心听取各民主党派、工商联、无党派人士意见。健全完善重大行政决策程序制度、合法性审查制度和集体讨论制度。贯彻落实中央八项规定精神，大力整治形式主义、官僚主义的十种新表现，驰而不息纠正“四风”。深入开展政府系统反腐败斗争，坚持无禁区、全覆盖、零容忍，坚持重遏制、强高压、长震慑，确保干部清正、政府清廉、政治清明。坚持以强化落实作为政府工作的主旋律。坚持领导带头、以上率下，敢于直面矛盾问题，善于破解棘手问题，以钉钉子精神抓落实，切实把各项决策部署落到实处。完善政绩考核评价体系，健全正向激励机制。落实容错纠错机制，旗帜鲜明地为担当者担当、为改革创新者撑腰鼓劲，让想干事的有机会、敢干事的有舞台、会干事的有作为。

各位代表，新时代赋予新使命，新时代呼唤新作为。让我们紧密团结在以习近平同志为核心的党中央周围，以习近平新时代中国特色社会主义思想为指导，在市委的坚强领导下，锐意进取，埋头苦干，为推动党的十九大精神在梧州落地生根、开创新时代梧州改革发展新局面而努力奋斗！
